# Reinforced Concrete Beam under Support Removal—Parametric Analysis

**DOI:** 10.3390/ma14205917

**Published:** 2021-10-09

**Authors:** Seweryn Kokot

**Affiliations:** Faculty of Civil Engineering and Architecture, Opole University of Technology, 45-758 Opole, Poland; s.kokot@po.edu.pl; Tel.: +48-77-449-8613

**Keywords:** reinforced concrete, progressive collapse, finite element analysis, support removal, vertical pushover, pushdown

## Abstract

This paper investigates the behaviour of a reinforced concrete beam under a support removal. A detailed parametric analysis is carried out, covering the effect of support removal rate on dynamic response. The linear elastic and nonlinear inelastic responses are computed and studied in detail. Critical parameters during the structural response are identified. In order to determine the ultimate load, the vertical pushover analysis is performed. The key parameters driving the beam response are assumed as random variables, and respective reliability study makes it possible to check the overall uncertainty of the dynamic response. In particular, the response spectrum measuring the effect of support removal rate has been computed. It has been demonstrated that the critical vertical response occurs when the time of support removal is up to to 17% of the first natural period. The vertical pushover analysis results in obtaining capacity curves and showed the order in which two plastic hinges occur for various load patterns. Finally, the reliability-based sensitivity analysis indicates the geometric cross-section cover and height are the most sensitive parameters of the beam response.

## 1. Introduction

The recent (24 June 2021) partial collapse of the Champlain Towers South condominium located at the Miami suburbs of Surfside gave rise to increasing attention of better understanding of progressive collapse of buildings. The research of this problem has a long tradition starting from the Ronan Point apartment building partial collapse in London 1968 [1]. The problem came back to the spotlight of engineers after the collapse of the Alfred P. Murrah Federal Building (Oklahoma City, 1995) (e.g., Osteraas [2] and Kazemi-Moghaddam and Sasani [3]) or World Trade Center collapse in 2001 (e.g., Bazant and Verdure [4]).

The progressive collapse phenomenon happens when a local failure causes successive structural damages leading to the partial or total collapse of a building. The problem of progressive collapse of buildings often leads to human life losses enhancing fear in the society and generating substantial economic costs. Therefore, further progress in understanding this phenomenon at various stages (in designing, erecting and maintaining buildings) is of great importance to mitigate its negative consequences. An extensive literature review on research and practice in the field of progressive collapse can be found in Mohamed [5], Nair [6], Kokot [7], Kokot and Solomos [8] and Adam et al. [9].

Since the reinforced concrete composite is one of the most popular materials used in structures, this paper is devoted to the behaviour of a reinforced concrete beam structure under a support removal. Nonlinear modelling of reinforced concrete structural members has already been analysed by the author of this paper using concentrated plastic hinges [10,11]. However, this study focuses on the distributed plasticity using the so-called fibre modelling of reinforced concrete cross-sections. Such more detailed, parametric analyses of structural response under support removal can be beneficial before planning complicated 3D finite element analyses of buildings in progressive collapse.

The behaviour of reinforced concrete structures is a challenging subject, not only in static analyses (e.g., Dudziak [12]) and dynamic earthquake engineering (e.g., Paulay and Priestley [13]) but also in dynamic analyses under accidental loads acting on structures (e.g., Ellingwood [14] and Grierson et al. [15]). In particular, the progressive collapse caused by excessive corrosion [16], earthquake loads [17,18] or bidirectional seismic effects [19], have been studied. At the material level, the reinforced concrete composite is a subject of extensive research in various areas such as the constitutive models, mechanical properties (e.g., Neville [20], Kent and Park [21], Scott et al. [22], Mander et al. [23], Galeota et al. [24]), the effect of strain-rates (e.g., Scott et al. [22], Mainstone [25], Asprone et al. [26], Filiatrault and Holleran [27], du Beton [28,29], Malvar and Crawford [30], Fu et al. [31], Bischoff and Perry [32], Malvar [33]), the assessment of concrete confinement [21,24,34,35,36,37,38,39,40] or strengthening of reinforced concrete with fibre-reinforced polymer materials (e.g., Derkowski and Walczak [41]). Moreover, there have been many advances in nonlinear behaviour of reinforced concrete frame structures, both in simulations and experiments (e.g., Kozielova et al. [42], Limkatanyu and Spacone [43], Kenyon and Warner [44], Filippou and Issa [45], Oller et al. [46]) as well as in assessing the robustness of reinforced concrete at various loading conditions (e.g., Praxedes and Yuan [47], Bao et al. [48], Mucedero et al. [49], Main et al. [50]).

The nonlinear inelastic modelling of reinforced concrete is based on the guidelines recommended in Bao et al. [51] and Alashker et al. [52] and is validated by reproducing the results of the vertical pushover curves in Bao et al. [51] where the authors reported the behaviour of reinforced concrete subassemblies modelled with micro- and macro-elements. The micro-elements are referred to continuum type finite elements (the model composed of such elements was considered as a high fidelity model), whereas the macro-elements are the beam or frame finite elements using various formulations (displacement-based or force-based elements; see e.g., Neuenhofer and Filippou [53], Calabrese [54]). According to Alashker et al. [52], in order to perform progressive collapse analyses effectively (in reasonable time and computer memory usage, preferably on personal computers), the modelling simplifications are inevitable; however, the degree of simplification must be controlled. The authors of this paper proved that a properly calibrated model using macro-elements can be regarded as reliable as far as accuracy of the material and structural behaviours are concerned.

Although there are many research studies on more complex structures, this paper focuses on a reinforced concrete two-span beam under a support removal. Such beams can represent, for example, footbridges or regular bridges.

The three objectives of this paper are: (1) the examination of the impact of support removal rate on the dynamic response, (2) the observation of the nonlinear behaviour of reinforced concrete beams under increasing deformation up to collapse (through the vertical pushover analysis using various load patterns), and (3) reliability-based sensitivity studies leading to the identification of the most important geometric and material parameters under vertical pushover loading conditions.

In the author’s opinion the original/novel contributions and findings of this paper are:

1. Establishing a characteristic shape of a response spectrum curve.
2. Comparison of various load patterns in the vertical pushover analyses and finding the percentages of the reduced ultimate capacities for concentrated load patterns compared to the uniformly distributed loading.
3. Identification of important input parameters (according to the so called importance measures) under the vertical downward loading as it is the case of progressive collapse.

The paper begins with Section 2 which introduces the computational model of the analysed beam. Section 3 is devoted to the determination of the response spectrum of the analysed beam for wide range of support removal rates. Section 4 deals with the vertical pushover analysis enabling us the assessment of the ultimate load and understanding the failure mechanism. Section 5 addresses the sensitivity studies using the the so called important measures of the first-order reliability method. Finally, Section 6 summarises and concludes this paper.

It should be emphasised that all models used in this paper have been created and analyses have been performed in the OpenSees free open-source software framework (OpenSees is a free software framework that enables users to create finite element applications for simulating the behaviour of structures under static and dynamic loading. It is an open source project started by Frank McKenna and other researchers joined to its developmentdeveloped by other researchers [55,56,57]. OpenSees users can create application using Tcl [58,59] and Python [60] scripting programming languages.

## 2. Computational Model of the Analysed Structure

**Figure 1:**
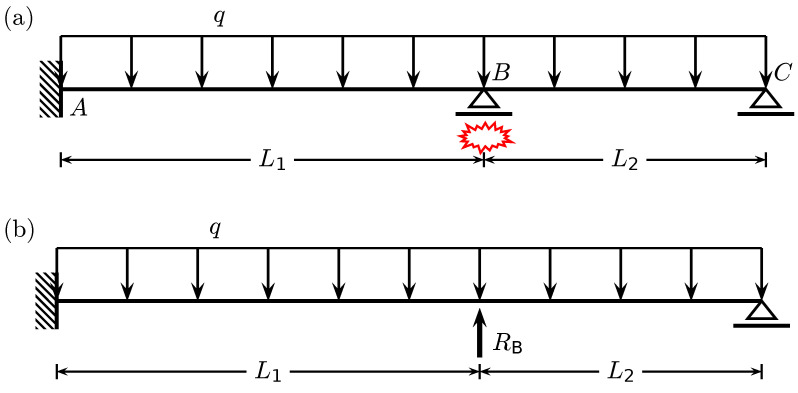
Computational model of (**a**) a two-span beam with the inner support removal scenario and (**b**) an equivalent single span beam with reaction $R_{B}$ to be removed.

Consider a two-span beam which a computational model is shown in Figure 1a. The beam has three supports: the left fixed support (node A), the inner roller one (node B) and the right roller support (node C). The lengths of the spans are $L_{1}=6\;m$ and $L_{2}=4\;m$. The beam is made of concrete with the initial modulus of elasticity $E_{c}=10.6\;\mathrm{Gpa}$, and the cross-section is rectangular for the whole beam. Assuming that the inner support is to be destroyed due to an unspecified accidental loading, the beam becomes a single span beam of length L=10m, when the reaction $R_{B}$ vanishes to zero (see Figure 1b).

**Figure 2:**
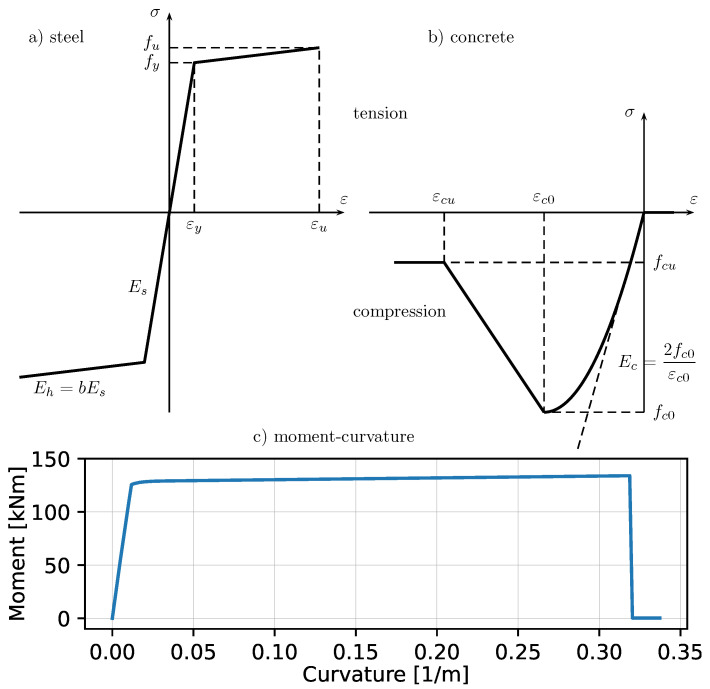
Backbone of the stress–strain relationships of concrete (**a**) and steel (**b**) and the moment–curvature for the cross-section of the two-span beam (**c**)—due to antisymmetry only the positive half is shown.

In the linear elastic analysis, the OpenSees ’elasticBeamColumn’ Euler–Bernoulli finite element is used, whereas for the nonlinear inelastic analyses, the ’forcedBeamColumn’ element is chosen. This force-based element is based on the formulation proposed and developed in Neuenhofer and Filippou [61] and Neuenhofer and Filippou [53], where the plasticity is controlled in five Gauss points located along the beam, according to the Gauss–Lobatto scheme, and the reinforced concrete cross-sections are modelled using the so called fibre modelling. To each fibre of discretised cross-section, a constitutive material relationship is assigned for both steel and concrete. The steel constitutive model is based on the Menegotto–Pinto formulation [62,63,64,65] (OpenSees ’Steel01’ uniaxial material model; see Figure 2a for the backbone of the steel stress–strain relationship). The concrete material model uses OpenSees ’Concrete01’ implementation, which neglects the concrete tensile capacity while in compression, is characterised by the initial parabolic stress–strain curve up to the ultimate strength $f_{c0}$ (e.g., Hognestad [66]), followed by linear descending branch, down to the residual stress $f_{cu}$. The backbone of the concrete stress–strain relationship is shown in Figure 2b. For beams, the cross-section is usually considered unconfined with zero residual stress. The inclination of the descending line depends on the amount and distance of transverse reinforcement. The theory behind this concrete material follows the works of Kent and Park [21], Hognestad [66], Karsan and Jirsa [67] and Scott et al. [22]. In the subsequent sections, in the nonlinear inelastic analyses, the following material parameters have been used: $f_{c0}=10.6\;\mathrm{Mpa}$, $\varepsilon_{c0}=0.002$, $f_{cu}=0.2\;f_{c0}$, $f_{y}=500\;\mathrm{Mpa}$ and $E_{s}=200\;\mathrm{Gpa}$, and the steel strain–hardening ratio *b* (ratio between post-yield tangent and elastic tangent) is assumed as 0.001. It should also be noted that in order to account for large displacements, the so called corotational formulation (e.g., De Souza [68], Crisfield [69]) is used in the nonlinear static and dynamic analyses.

The cross-section is designed for the uniformly distributed loading of 5kN/m, resulting in the rectangular concrete section: 0.2m wide and 0.4m high. The longitudinal reinforcement represents 3ϕ16 bars in the bottom and 3ϕ16 in the top (symmetry about the horizontal neutral axis). The cross-section is modelled and analysed in OpenSees and its moment-curvature characteristics are shown in Figure 2c. The first steep increasing segment (meaning the increase both in concrete stress of the compressed zone and in axial force of the tensile rebars) ends with the onset of yielding of the tensile rebars at the bending moment of 125kNm. Then, in the second segment, the concrete stress further increases up to the compressive strength $f_{c0}$, and at the same time, the compressed concrete zone moves up the cross-section until the strain of tensile rebars exceeds the fracture limit, which happens at the ultimate curvature of about $0.32\;m^{-1}$. Due to symmetry of the cross-section, the moment–curvature curve is antisymmetric, and only the right side of the plot is shown. The typical shape of the moment–curvature curve agrees with experimental tests for rectangular reinforced concrete beams (see, e.g., Park and Paulay [70], Espion and Halleux [71], Srikanth et al. [72]).

**Figure 3:**
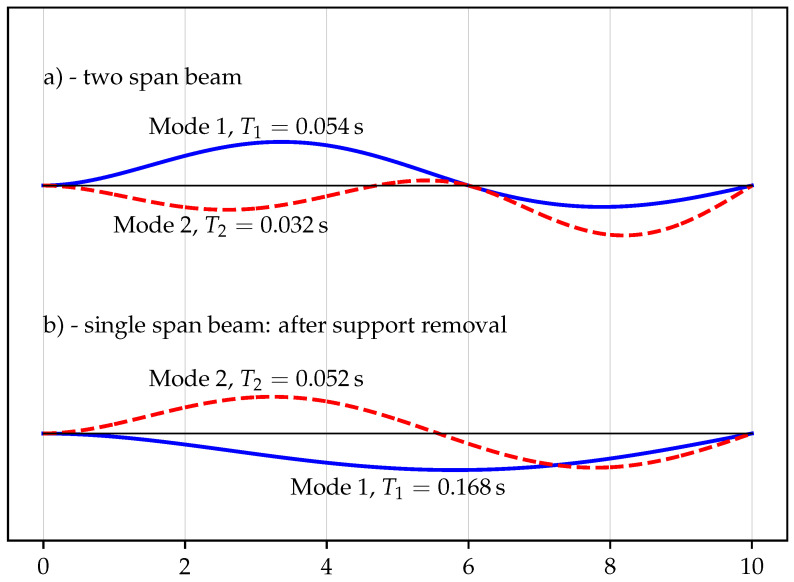
Mode 1 and 2 of the beam (**a**) before inner support removal and (**b**) after inner support removal.

In dynamic analyses, it is valuable to perform the modal analysis to get information on dynamic properties of the structure, and therefore, first, the eigenvalue problem is solved for the initial two-span beam. The first two natural periods are equal to $T_{1}=0.054\;s$ and $T_{2}=0.032\;s$, and their associated mode shapes are presented in Figure 3a.

Since the inner support is to be removed, it is even more important to get the dynamic characteristics of a single span beam (without the inner support). In this case, the first natural period is equal to $T_{1}=0.168\;s$ (more than three times longer than for the initial model), and the second one is $T_{2}=0.052\;s$ (1.6 times longer than previously). Obviously, the natural periods are greater in the second case due to smaller stiffness of the resulting one span fix-roller support beam. The first two mode shapes are illustrated in Figure 3b.

It should be noted that although for beams, the mode shapes and vibrations are only in the vertical direction, for frames, often the dominating motion (first mode shape) is in the horizontal direction. Therefore, the modal analysis should mainly identify the modal characteristics in the vertical (gravitational) direction when analysing the progressive collapse problem.

## 3. The Impact of the Sudden Support Removal

Assuming that the computational model of the structure is adapted to the formulation of the finite element method, for the nonlinear inelastic model, the following matrix equation of motion needs to be iteratively solved

(1) $$M\ddot{U}+P_{r}\left(U,\dot{U}\right)=P\left(t\right)$$

where M is the mass matrix, $\ddot{U}$ is a vector of accelerations, $P_{r}\left(U,\dot{U}\right)$ is the vector of nodal resisting forces (which can be linearised using the Newton–Raphson method), and P(t) is the vector of external time-dependent loads. When the model is linear elastic, the above equations can be simplified to

(2) $$M\ddot{U}+C\dot{U}+KU=P\left(t\right)$$

where C and K are the damping and stiffness matrices, whereas $\dot{U}$ and ***U*** are the vectors of velocities and displacements, respectively. Equations (1) and (2) can be numerically solved using direct time integration methods such as Newmark family or Hilber–Hughes–Taylor (HHT) methods (e.g., Newmark [73] and Hilber et al. [74]). In this study, the Newmark method is used as implemented in the OpenSees finite element method framework.

In Section 3.1 and Section 3.2, the dynamic behaviour of the two-span beam under sudden (shortest possible duration) and increasing durations of support removal is investigated.

### 3.1. The Sudden Support Removal

First, let us begin with the analysis of the most adverse dynamic response. To this end, assume that due to an unspecified cause, the inner support is suddenly destroyed. Therefore, the support removal time $t_{r}$ is equal to the time interval dt=0.001s used in the Newmark integration scheme when solving the system of the equations of motion. It is also assumed the so called Rayleigh damping is in the form of αM+βK where the parameters are proportional to the mass and stiffness matrices. Given 5% damping ratio for the first two modes and using the formula $\xi_{i}=\frac{\alpha }{2\omega_{i}}+\frac{\beta \omega_{i}}{2}$, it results in α=2.3 and β=0.000162.

In any finite element method software which offers a module for the solution of linear or nonlinear dynamic problems, the sudden support removal can be modelled in two stages. In the first stage, the support to be removed is included in the model, and after applying the gravity loads, the reaction values need to be recorded. In this case, the value of $R_{B}$ at support B must be noted. In the second stage, in the other modified model, the support (not present) is replaced by the reaction force to obtain a model equivalent to the initial one. Then, a decreasing in time force (of the initial value equal to the support reaction and the downward direction) is applied. In this way, we can control the time in which the reaction is cancelled and hence simulate instantaneous support removal.

**Figure 4:**
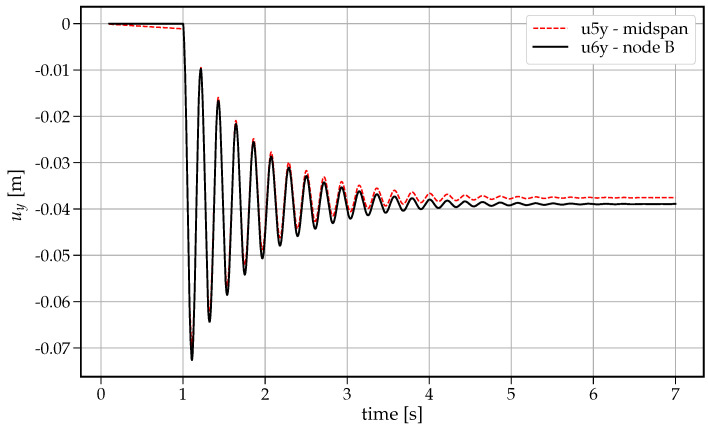
Time history of vertical displacements at midspan ($u_{5y}$) and at node B ($u_{6y})$.

The following procedure is implemented, and the analysis is performed in OpenSees software framework. To this end, a uniformly distributed load is statically applied within 1 s. Then, at time 1.001 s, the reaction $R_{C}$ is cancelled by counter-reaction $-R_{C}$. The beam begins to deform downward, reaching the maximum deflection 0.072595 s at time 1.110s, following by the free damped vibration stage as illustrated in Figure 4. The vertical displacements at two characteristic nodes are shown: midspan node at distance x=5m from the left fixed support and node B (x=6m) of the removed support (see Figure 1). Due to different types of supports (the left support is fixed, and the right one is a roller support) a slightly larger displacement is developed at node B.

**Figure 5:**
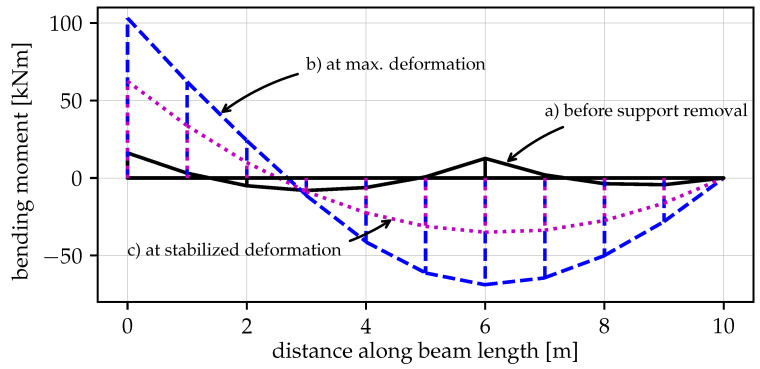
The evolution of the bending moment distribution—(**a**) initial model with the inner support (at time 1.00s), (**b**) at maximum deformation after sudden inner support removal (at time 1.11s), (**c**) without the inner support–situation at stabilised deformation (at time 7s).

Figure 5 shows the evolution of the bending moment distribution for the whole process of the sudden inner support removal. Figure 5a presents the bending moment diagram of the model with the support B and after applying the gravity load. The largest bending moment is at support B, M=16.177kNm. In turn, Figure 5b,c displays the bending moment distributions (after the removal of the inner support) at the time of maximum vertical displacement (1.110s) and at the final stabilised position (7s), respectively. Comparing the maximum and stabilised bending moments leads to dynamic factors of 103.219/62.5=1.65 at the fixed support A and 68.948/35.0=1.97 at node B (x=6m).

### 3.2. The Response Spectrum to Various Support Removal Rates

**Figure 6:**
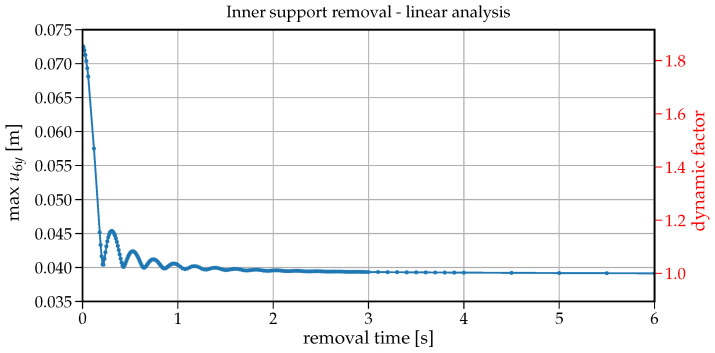
Plot of support removal time $t_{r}$ versus maximum vertical displacement at node B–linear analysis.

**Figure 7:**
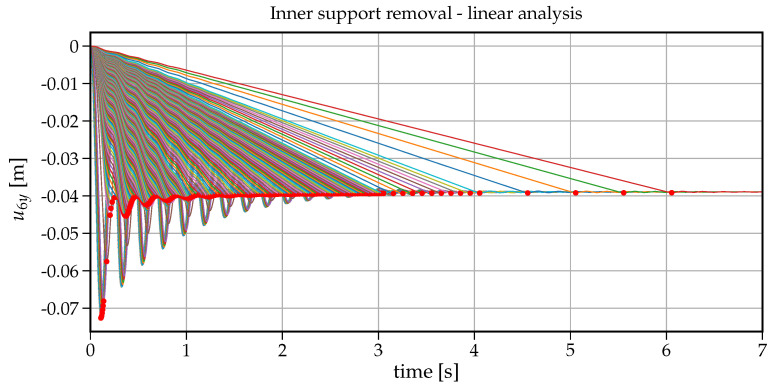
Time history plots of the vertical displacement at node B for all support removal times $t_{r}$–linear analysis.

**Figure 8:**
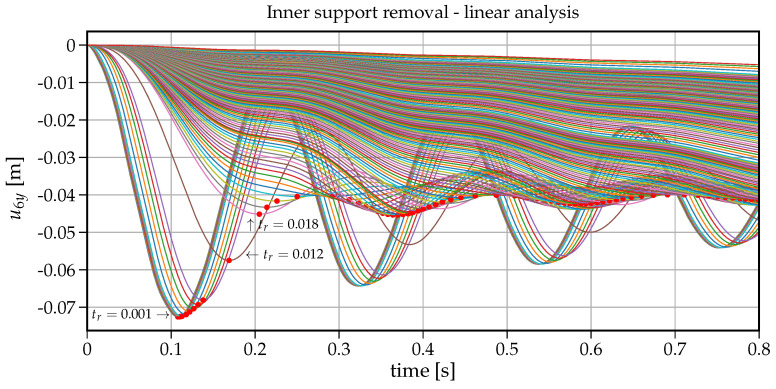
Enlarged Figure 7–linear analysis.

To analyse the impact of the support removal time on the dynamic response (here the vertical displacement) at node B of the removed support, a set of analyses have been run for removal times $t_{r}$ changing from 0.001s to 6s. Figure 6 shows how the maximum vertical displacement $u_{6y}$ at node B changes with the increasing values of $t_{r}$. On the right-hand side in this plot, there is also information on the dynamic factor in reference to the static displacement (that is for the case when the support is removed within $t_{r}=6\;s$). The values collected in Figure 6 are extracted from individual 75 time histories shown in Figure 7. The plot in Figure 6 can also be regarded as a response spectrum of a structure undergoing the support removal for a wide range of removal durations. To have some insight into the development of the first crucial part of vibrations, an enlarged version of Figure 7 is presented in Figure 8. Both in Figure 7 and Figure 8, the red dots indicate the maximum downward displacements collected in the response spectrum of Figure 6.

Note that the maximum vertical displacement of $u_{6y}$ is obtained for $t_{r}$ containing in the range of 0.001−0.01s. For $t_{r}=0.001\;s$, the maximum vertical displacement equals $u_{6y}=0.0726\;m$ and for $t_{r}=0.01\;s$, $u_{6y}=0.0724\;m$ with only 0.2% relative difference. For $t_{r}=0.052\;s$, $u_{6y}=0.069\;m$, the relative difference increases to 5%. It means that sudden support removal can be considered for up to $t_{r}=0.052\;s$, which is 30% of the first natural period ($T_{1}=0.168\;s$). With further increase in $t_{r}$, we can observe a fast decrease in the maximum vertical displacement down to the point of $(t_{r}=0.168\;s$, $u_{6y}=0.040\;m)$. The ordinate of this point coincides with the fundamental period of beam after the support removal (see Figure 3b). Following further the curve in Figure 6, there are six noticeable cycles where alternately the maximum vertical displacement increases and decreases.

**Figure 9:**
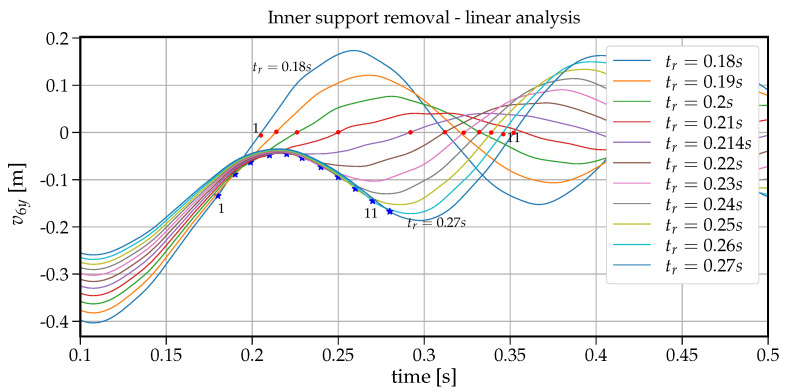
Time history plots of the vertical velocities at node B for selected support removal times $t_{r}$–linear analysis.

At first glance, it seems counter-intuitive that for greater values of $t_{r}$, the maximum vertical displacement is greater than for a smaller $t_{r}$. However a closer look at the associated velocity values helps understand this phenomenon. The related time instants where the vertical displacement takes the maximum value are shown in Figure 9 with dots whereas asterisks depict the times when the cancellation of the support reaction ended. In this way, the velocities indicate that (a) the maximum vertical displacement occurs when the respective velocity is zero (dots in Figure 9), (b) when $t_{r}$ exceeds the period $T_{1}=0.168\;s$, the second cycle of vibration begins in the downward direction and hence delays approaching the value of zero velocity. The length of these cycles is of about 0.17s and coincides with the first natural period. Eventually with longer support removal times ($t_{r}>2.5\;s$), the maximum vertical displacement stabilises and converges to the static displacement of 0.039m.

It should also be noted that the maximum dynamic factor reaches the value of 1.8 (see Figure 6).

**Figure 10:**
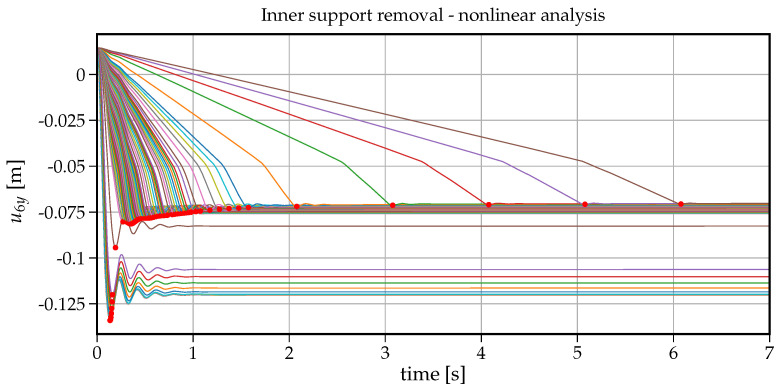
Time history plots of the vertical displacement at node B for all support removal times $t_{r}$–nonlinear analysis.

A similar study can be carried out when the material properties for concrete and steel are nonlinear inelastic. Let us start with the individual time-histories of the vertical displacement at node B versus the increasing support removal times $t_{r}$ as shown in Figure 10. For very short removal times ($t_{r}<0.06\;s$), the beam response is highly inelastic, and after the first waves of vibrations, a significant plastic deformation is noticeable. Moreover, for all individual time-histories, the duration time of the free vibration is shorter than in the case of linear analysis, which can be explained by the fact that, in the nonlinear case, the tangent stiffness changes in different phases of deformation, and this fact can be regarded as an additional damping factor.

**Figure 11:**
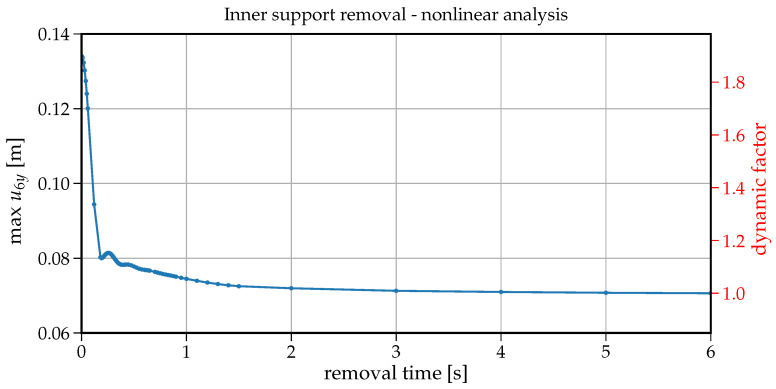
Plot of support removal time $t_{r}$ versus maximum vertical displacement at node B–nonlinear analysis.

The maximum values of the individual 75 time-histories shown in Figure 10 are collected and presented in the form of the inelastic response spectrum in Figure 11 where the maximum vertical displacement $u_{6y}$ at node B is plotted versus the increasing values of $t_{r}$. On the right of this plot, we can see the dynamic factor scale in reference to the static displacement. The maximum dynamic factor reaches the value of 1.91 for $t_{r}=0.005\;s$ and quickly decreases to the values of 1.1–1.2 for $t_{r}=0.20\;s$; then, the maximum displacement of $u_{6y}^{\max}$ slowly approaches the static displacement. It is interesting that the characteristic cycles visible in the linear elastic response spectrum (see Figure 6) are not present here, mainly, because the natural period changes in time as the nonlinear inelastic vibrations proceed.

It should be emphasised that in the nonlinear case, the structural response depends on the excess of capacity with which a structure is designed against a particular support or other load-bearing element removal scenario. Would there be no redundancy in the capacity, the structure would not survive the first downward movement stage leading to partial or total collapse.

It could be argued that the present study contains the systematic assessment of the influence of support removal rates on the dynamic response. Existing publications (e.g., Russell et al. [75,76] and Adam et al. [77]) usually consider selected column removal durations for specific assumed fast-dynamics failure events (e.g., blast), but simulations or experimental tests of slower-dynamics element failures such as impacts or material deterioration require longer load-bearing element removal duration. In this light, this analysis gives further insight into the impact of support/column removal rates on the structural dynamic behaviour which, in turn, can be helpful in planning expensive experimental tests and/or designing an appropriate support/column removal technique.

## 4. The Vertical Pushover Analysis

Now, let us perform a vertical pushover analysis of the two-span beam to evaluate its ultimate capacity against the inner support removal. It should be noted that the initial system is twice statically indeterminate which means that formation of the second plastic hinge transforms the system into a mechanism, and the structure can be regarded as collapsed. From the numerical point of view (and the finite element method) the onset of a mechanism can be indicated by the lack of numerical convergence.

**Figure 12:**
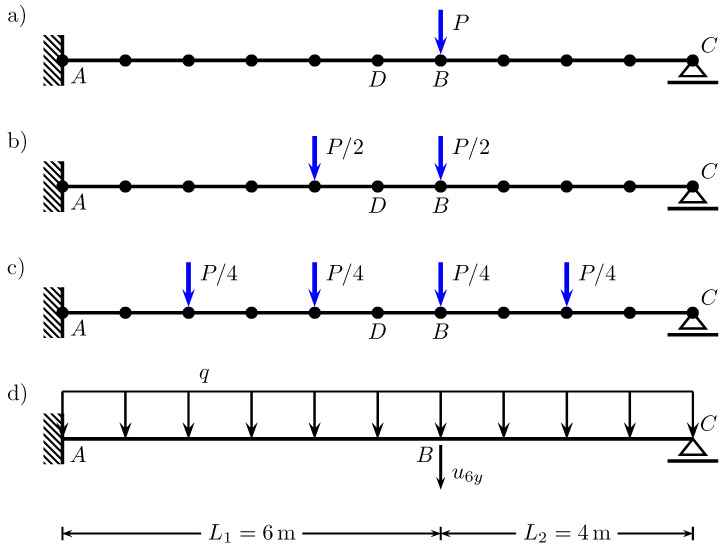
Four cases of applied loading distribution in vertical pushover analyses: (**a**) only one vertical force *P*, (**b**) two forces P/2, (**c**) four forces P/4, (**d**) uniformly distributed load q=P/L.

According to the theory of pushover analysis in the seismic engineering, the reference loads and controlled displacement are considered in the horizontal direction. Here, in the progressive collapse problems, the pushover analysis can be adapted for the vertical direction when the support is removed from the model and the vertical deformation is induced until reaching the ultimate capacity of the system. The reference vertical loads are applied to the model in the displacement control strategy to reach a prescribed target displacement at a chosen node and vertical degree of freedom. In many experimental tests (e.g., Lew et al. [78,79]), one concentrated force is applied to the structure mainly to simulate the inertia forces generated by sudden removal of a load-bearing element. One concentrated force is selected due to simplicity reasons; however, in realistic progressive collapse events in smaller frames, a uniformly distributed gravity load can better represent the real external force distribution. Therefore, in the following considerations, four cases of applied loading distribution are used. The assumption is that in all cases, the sum of vertical loads is equal to the reference force P=1kN. This is the basic simplified case shown in Figure 12a. In the second and third cases, the reference loading is represented by two P/2 and four P/4 forces, respectively, as illustrated in Figure 12b,c. The last case is the realistic load pattern in the form of the uniformly distributed load q=P/L (see Figure 12d).

The main outcome of the vertical pushover analysis is the capacity curve which represents the total vertical loading (hence the sum of vertical support reactions) as a function of the increasing vertical displacement $u_{6y}$ at the point of the removed support.

**Figure 13:**
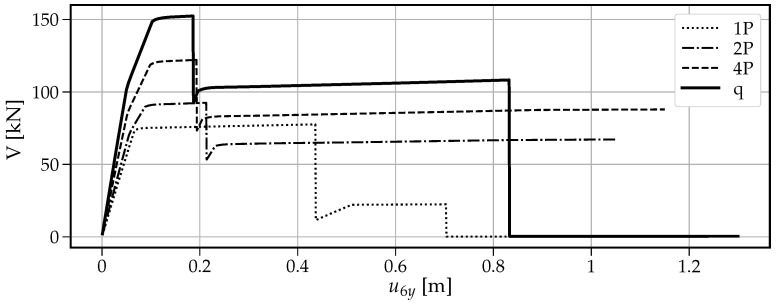
The vertical load *V* as a function of the vertical displacement $u_{6y}$ of a two-span beam.

**Figure 14:**
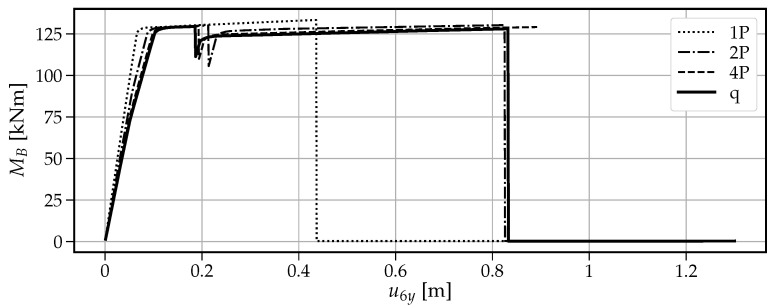
Evolution of the bending moment $M_{B}$ with the increasing vertical displacement $u_{6y}$.

**Figure 15:**
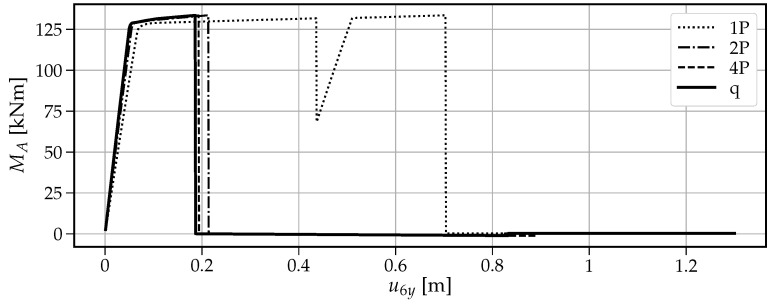
Evolution of the bending moment $M_{A}$ with the increasing vertical displacement $u_{6y}$.

**Figure 16:**
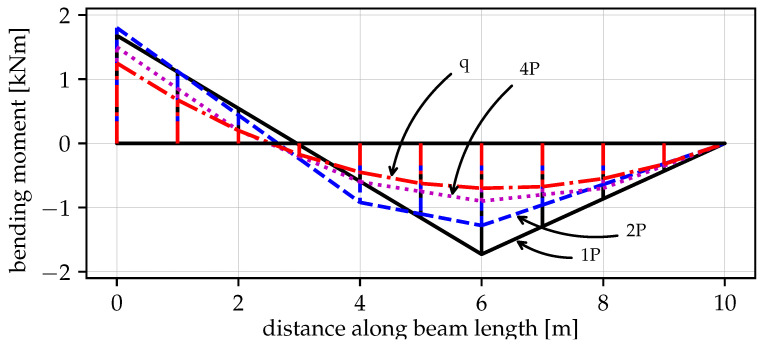
Bending moment distribution of the beam after the inner support removal for 1, 2, 4 concentrated forces *P* and uniformly distributed force *q*.

Figure 13 shows that when a single vertical force “1P” is applied, the maximum vertical load the beam can withstand is the smallest value of V=77.6kN among the four cases. The first drop at $u_{6y}=0.436\;m$ is caused by the formation of the first plastic hinge at point B. This fact is visible in Figure 14 where at the same time instance, the moment capacity drops to zero. However, there still exists some residual vertical capacity of about (V=22.4kN) up to $u_{6y}=0.703\;m$, where the second plastic hinge at point A causes the moment $M_{A}$ drop to zero (see Figure 15) and eventually transform the beam into a mechanism. The order of plastic hinges, in which they develop, is associated with the bending moment distribution presented in Figure 16 (which shows the bending moment diagram for a unit force). It can be seen that the maximum bending moment is located at point B and is equal to 1.728, compared to the smaller value of 1.68 at point A. The same diagram indicates that for other load pattern cases: “2P”, “4P”, and “q”, the maximum bending moment is located at point A, while the bending moment at point B is smaller. The relevant values for $M_{A}$ are: 1.8, 1.5, 1.25 and for $M_{B}$: 1.28, 0.8, 0.7, respectively. This observation will imply the order of plastic hinges in which they occur in the “2P”, “4P” and “q” cases of the vertical pushover analysis.

**Figure 17:**
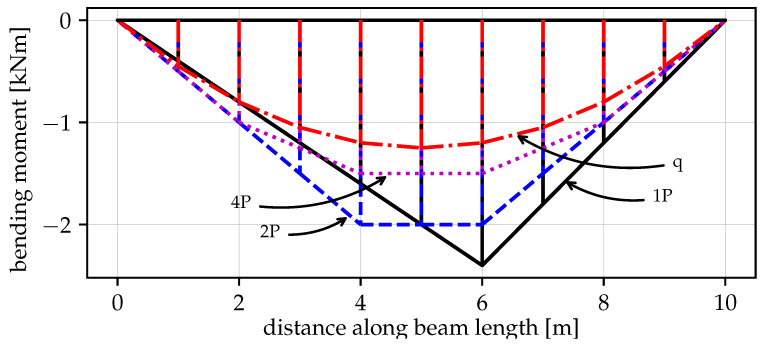
Bending moment distribution of the beam after the inner support removal and after formation of the plastic hinge at the left fixed support (now pinned support) for 1, 2, 4 concentrated forces *P* and uniformly distributed force *q*.

**Figure 18:**
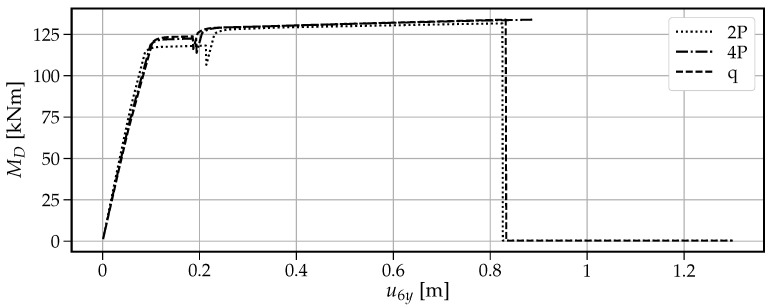
Evolution of the bending moment $M_{D}$ with the increasing vertical displacement $u_{6y}$.

Moreover, Figure 13 shows that when the load is distributed over a wider span, then the vertical capacity increases: for “2P”: $V^{\max}=91.8\;kN$; for “4P”: $V^{\max}=122.1\;kN$ and for “q”: $V^{\max}=152.4\;kN$. On the other hand, the more vertical load is distributed, the earlier the first hinge develops ($u_{6y}$: 0.216m, 0.194m and 0.187m for the “2P”, “4P” and “q” load patterns, respectively); however, this difference is not significant. Then, upon reaching the maximum vertical capacity, the beam in these three cases is transformed into a simply supported beam, which in turn modifies the bending moment distribution as presented in Figure 17. Now, the next plastic hinge is expected to occur at the point of the maximum bending moment which is the midspan of the simply supported beam (point D). In fact, Figure 18 shows that at $u_{6y}=0.832\;m$, the moment $M_{D}$ drops to zero, indicating the formation of the plastic hinge at point D. Figure 13 shows that up to this point, the beam for the “q” case, still demonstrates significant vertical load capacity of 108.2kN (which is greater than the maximum capacities for the “1P” and “2P” load patterns. When it comes to the “2P” and “4P” cases, the computations have been stopped due to lack of convergence, which means that the last successful pseudo-time step takes place right before the occurrence of the second plastic hinge at point B and the resulting instability caused by the mechanism preventing convergence. Looking at Figure 17 can lead to the conclusion that the lack of convergence can also be caused by simultaneous formation of multiple plastic hinges because for the “2P” and “4P” cases, the maximum bending moment is not located at one particular point but spread over the distance of two meters (x∈〈4−6m〉). This situation seems to be similar to the buckling bifurcation or snap-through issues encountered in frame or shell structures.

From the physical viewpoint, it is interesting to compare the obtained capacity curves to the vertical pushover curves reported in many studies on the widespread subassembly (a two-span beam with two side columns and the removal of the central column) (e.g., Bao et al. [51], Alashker et al. [52], Lew et al. [78,79] Bao et al. [80]). This subassembly is considered as an extracted fragment of larger multi-span, multi-storey plane frames and is useful as a specimen for experimental tests. It can be summarised (according to Bao et al. [51]) that the capacity curve in such a subassembly is characterised by three stages: (1) the ascending stage responsible for the concrete arch effect, (2) the descending one indicating the development of a plastic hinge and finally (3) the second ascending stage due to the so called catenary action which ends with the collapse. In the first stage, the axial forces in the beams are in compression because the side columns resist the outward movement. However, in real frames, where many bays and storeys are involved, the outward movement of columns can be negligible. From the reinforced concrete cross section viewpoint, in this stage, the concrete cracking in the tensile zone is developed, and the steel bars exhibit increase in both tensile and compressive forces. The descending stage is responsible for further increase of concrete compressive stress up to crushing in the extreme fibres, and the cracks in the tensile zone continue to grow. The side columns gradually move back towards the initial position. In the second ascending stage, the catenary action develops because of (1) further growth of cracks, (2) change of rebar forces in the initial compressive zone from compressive to tension and (3) inward movement of side columns. As showed in Bao et al. [51], Alashker et al. [52] and Bao et al. [80], the realistic physical phenomena described above were both represented in both micro and macro finite element models, which proves that the macro finite elements are effective in reflecting the physical behaviour of reinforced concrete beams or columns under progressive collapse loading conditions. Finally, the collapse can happen due to, for example, the pull-out of rebars from the anchorage zones or the fracture of rebars (depending on the detailing). The numerical simulations and the described phenomena have been confirmed in full scale experimental tests ( e.g., Lew et al. [78,79] and Bao et al. [80], Gu et al. [81]).

In the light of the above, the ascending stage in Figure 13 is characterised by segments of lines with decreasing inclination because each of the two critical cross sections separately enters the hardening part of the moment–curvature relationship (see Figure 2). The arch effect (reflected by the compression in beams) is not present, here, because the boundary condition (the right roller support) does not impose any lateral resistance. In fact, in this stage, the axial force in beam (near midspan) is close to zero, and the horizontal displacement of the roller support exhibits a small negligible value to the right. Then the descending stage is abrupt, signifying that one of the critical sections reached the ultimate curvature due to the tensile rebar fracture (see Figure 2c), and the resulting capacity depends only on the other critical cross section. Finally, in the second ascending stage, the concrete cracking increases, and the catenary action is reflected by large vertical displacement and the slowly increasing horizontal displacement to the left. Looking at the forces in beams and horizontal displacement of the roller support reveals that in the re-ascending stage of Figure 13, the axial force in beam takes relatively small values of 0.3–0.8 kN, and the horizontal displacement of the roller support increases to 0.12m when the vertical displacement $u_{6y}$ reaches 0.83m (the second plastic hinge and rebar fracture). It should be noted that without using the corotational formulation taking into account the geometric nonlinearity, both the arch effect and catenary action would be unidentifiable.

The insight from this two-span beam example can be helpful in understanding more complex frame structures, where yielding and plastic hinges are expected at several locations up to the point where the structure becomes unstable.

It should also be noted that in the many experimental tests (e.g., [78,79,81]), the increasing deformation is carried out by pushdown techniques concentrated at one node (the location where the removed column is connected to the remaining structure). Therefore, the current study sheds some light on potential underestimation in the ultimate capacity when performing the vertical pushover test. For practical reasons, it can be difficult to control deformation using actuators in many directions; hence, if only one node is loaded, the underestimation due to simplified load pattern should be taken into account.

Since this study focuses on beams, it is of interest to perform similar investigations pertaining to various configurations of frame structures (including other models of frame subassemblies) by applying realistic distributed load patterns.

## 5. Sensitivity Study

During the modelling stage, the values various material and geometric parameters (e.g., concrete compressive strength, concrete strain at peak stress, tensile concrete strength, concrete softening properties, steel yield and ultimate stress, uncertainty in final cross-section width, height and cover executed on building site, etc.) are assumed. These assumptions are due to limited knowledge or due to the inherent randomness of concrete or steel mechanical properties. Small changes in those parameters can produce significant unforeseen changes in local or global structural response. Therefore, it is valuable to perform sensitivity analysis to gain insights into the importance of parameters used in this study.

The calculation of sensitivity of a structural response with respect to uncertain properties indicates those parameters which have a significant influence on the structural behaviour. The sensitivity can be determined calculating the gradient of the structural response using the finite difference method or through direct differentiation with regards to parameters, which are included in the computational model of the structure and the applied loads. It is of utmost importance to identify parameters when their small change causes meaningful changes in structural response.

The structural response obtained using the finite element method depends highly on the assumptions of the model properties imposed in the analysis. These properties, specified by the parameters, describe the material, the finite elements it is made of, and the applied loads to the structure as well as the location of nodes. The parameters which describe the structural system can be collected in a vector x, and then, the equilibrium equation takes the form

(3) $$P_{r}\left(U\left(x,t\right),x\right)=P_{f}\left(x,t\right)$$

Both the vector of nodal forces $P_{r}$ and the vector of external forces $P_{f}$ depend on the parameter, explicitly. Additionally, the vector of nodal forces can depend implicitly on the parameters through the vector of nodal displacements ***U***.

There are basically two approaches to calculate the gradient ∂U/∂x of nodal displacements with respect to changes in a parameter *x*. The first approach is based on the direct differentiation method, where the equations of the system are differentiated analytically [82,83,84] whereas the other approach utilises the finite difference method, in which the analyses are repeated with a small change (perturbation) of the parameter x+Δx. Thus the gradient of the response can be evaluated as the difference between the response with and without the parameter perturbation divided by the parameter change

(4) $$\frac{\partial U}{\partial x}\approx \frac{U(x+\Delta x)-U(x)}{\Delta x}$$

It seems obvious that the direct differentiation method is more effective than the calculation based on the finite difference approach because the gradient of the response is obtained during a single analysis. Furthermore, the round-off errors are avoided, and no additional analyses with changes in parameter values are necessary. The detailed derivation of all required quantities in force-based element formulation both for linear and nonlinear geometry can be found in Scott [83], Scott et al. [84], Haukaas and Scott [85] and Scott [86].

Although the calculations of response sensitivities using the direct differentiation method or finite difference method can help understand what are the parameters raw sensitivities to a specified fixed parameter changes, for example, ±10% change, parameters have different accuracy in estimation which in turn leads to probabilistic approach, where they can be treated as random variable variables with a specified probability distribution function and its related mean and standard deviation characteristics. Therefore, in the following section, another approach, based on a particular reliability method, is briefly presented, and the notion of importance measures is presented after [87,88,89,90].

### 5.1. Sensitivity Based on the Theory of Reliability

Apart from the raw sensitivity methods, the theory of reliability ([89,91,92,93,94,95,96]) revealed other approaches to identifying which input parameters play a significant role in the structural response. To present the important measures which help identify the parameters contributing the most to the structural response, a brief introduction to reliability theory is needed. The considerations are narrowed to the class of time-invariant reliability problems.

Due to the well established literature notation of the standard normal random variable as a lowercase *u*, exceptionally in this section, a lowercase vector u stands for the vector of standard normal random variables and not the vector of element displacements as in earlier sections. However, still in this section, the uppercase ***U*** refers to the global displacement vector.

The structural reliability problem in the case of a single limit-state function (the so called component problem as opposed to the series, parallel and general system problems) can be formulated as *n*-fold integral

(5) $$p_{f}=\int \ldots \int_{g\leq 0}f\left(x\right)dx$$

where $p_{f}$ is an unknown probability, *g* is a limit-state function (also called a performance function) which determines the state of failure (unsafe state) for which the probability is to be found, and f(x) is the joint probability distribution function of random variables which constitute an *n*-dimensional vector x. In the reliability analysis using the finite element method, the limit-state function can be defined with respect to the response quantities, obtained from the finite element analysis. In engineering practical applications, the closed-form solution of Equation (5) is very difficult to obtain, and therefore, the approximate solution can be found using various methods such as the First-Order Reliability Method (FORM), the Second-Order Reliability Method (SORM) or Monte-Carlo techniques. The thorough descriptions of these methods can be found in Ditlevsen and Madsen [93], Der Kiureghian [97] and Chang [98]. In particular, the FORM method seems to be appealing because it requires only a few numbers of limit-state function evaluations during the finite element method analyses. Additionally, the FORM method provides valuable information on the significance of particular random variables which represent material or geometric parameters of the computational model.

In the FORM method, after transforming random variables x to the uncorrelated standard normal random variables u=u(x) and the limit-state function *g* to $g_{u}$ in the standard normal space, the probability in Equation (5) can be approximated as

(6) $$p_{f}=\int \ldots \int_{g_{u}\leq 0}\varphi \left(u\right)du$$

where φ(u) is the standard normal joint probability density function and the boundary of the limit-state function $g_{u}=0$ is approximated in the transformed standard normal space. When the limit-state function is nonlinear, the desirable approximation point is the point on the surface $g_{u}=0$, whose location is the nearest to the origin of the standard normal space. Such a point is called the design point $u^{*}$, and results from the solution of the following minimisation problem

(7) $$u^{*}=arg min\left\{∥u∥\;\mathrm{subject}\;\mathrm{to}\;g_{u}=0\right\}$$

where ‘arg min’ is the argument of a function at which the function is minimised.

When $g_{u}\left(u^{*}\right)=0$, the linearised limit state function can be expressed as

(8) $$g_{u}\left(u\right)\approx g_{u1}\left(u\right)=\nabla g_{u}\left(u^{*}\right)\left(u-u^{*}\right)=∥\nabla g_{u}\left(u^{*}\right)∥\left(\beta -\alpha u\right)$$

where $\nabla g_{u}\left(u\right)$ is the gradient row vector, $\alpha =-\nabla g_{u}\left(u^{*}\right)/∥\nabla g_{u}\left(u^{*}\right)∥$ is the normalised negative gradient vector at point $u^{*}$, and β=αu is the reliability index.

Gradients of the limit-state function $\nabla g_{u}=\partial g_{u}/\partial u$ are often used in the most effective algorithms which enable us to solve this optimisation problem. The differentiation of the limit-state function with respect to u can be expressed as

(9) $$\frac{\partial g_{u}}{\partial u}=\frac{\partial g_{u}}{\partial U}\frac{\partial U}{\partial x}\frac{\partial x}{\partial u}$$

where ∂x/∂u is the Jacobian of the probability transformation, ∂U/∂x stands for the gradients of the global structural response vector ***U*** with respect to parameters x, and finally $\partial g_{u}/\partial U$ is the derivative of the limit-state function with respect to the response vector ***U***.

The exchange of information between the reliability algorithm and the module of the finite element software consists in updating the finite element model by the realisation of random variables x and returning the global response vector ***U*** and its derivative ∂U/∂x to evaluate the limit-state function. Note that this technique has already been implemented and available in the OpenSees software framework Haukaas and Der Kiureghian [89], Scott and Haukaas [99].

After finding $u^{*}$, the probability $p_{f}$ in the FORM method can be approximated using the following formula

(10) $$p_{f}=\Phi \left(-\beta \right)$$

where Φ stands for the standard normal cumulative distribution function, and β is the reliability index (see, e.g., Haukaas and Scott [85], Ditlevsen and Madsen [93]).

In civil engineering structure applications, typically, the marginal distribution of random variables and their correlation coefficients are relatively straightforward to identify (see, e.g., Benjamin and Cornell [100]). The most effective probability transformation of non-normal random variables x into the standard normal random variables u is the Nataf transformation [90,91,101,102]. The Nataf model can be characterised by two desirable features: (1) the model can take into account broad span of correlation values between the random variables x, and (2) the transformation to standard normal space is relatively straightforward regardless of the order of random variables.

In the Nataf transformation, the joint normal distribution f(x) can be calculated from the marginal probability distribution functions $f(x_{i})$ and the matrix of correlation coefficients *R* as follows

(11) $$f\left(x\right)=f\left(x_{1}\right)\ldots f\left(x_{n}\right)\frac{\varphi_{n}\left(Z,\bar{R}\right)}{\varphi \left(z_{1}\right)\ldots \varphi \left(z_{n}\right)}$$

where $\varphi_{n}\left(z_{n}\right)$ is an *n*-dimensional joint probability distribution function with zero mean value, unit variance and correlation matrix $\bar{R}$. The term $\varphi (u_{i})$ is the standard normal probability distribution. The reciprocal relationship (e.g., Der Kiureghian [90], Liu and Der Kiureghian [91], Gu [96]) of a random variable $x_{i}$ by an arbitrary probability distribution function to a random variable $z_{i}$ (characterised by the standard normal distribution) can be expressed as

(12) $$z_{i}=\Phi^{-1}\left(F_{i}\left(x_{i}\right)\right)$$

The terms $F_{i}\left(x_{i}\right)$ and $\Phi (u_{i})$ stand for the cumulative distribution function of variables (of distribution $f(x_{i})$) and standard normal probability density function $\varphi (u_{i})$. $\bar{R}={\bar{\rho }}_{ij}$ while ${\bar{\rho }}_{ij}$ and $\rho_{ij}$ are related together by the following expression

(13) $$\rho_{ij}={\;\int \int \;}_{-\infty }^{\infty }\left(\frac{x_{i}-\mu_{i}}{\sigma_{i}}\right)\left(\frac{x_{j}-\mu_{j}}{\sigma_{j}}\right)\varphi_{2}\left(z_{i},z_{j},{\bar{\rho }}_{ij}\right)dz_{i}dz_{j}$$

where ${\bar{\rho }}_{ij}$ are the correlation coefficients between $z_{i}$ and $z_{j}$, and $\varphi_{2}\left(z_{i},z_{j},{\bar{\rho }}_{ij}\right)$ is the normal distribution function of a two-dimensional random variable with zero mean value and unit variance. In the iterative process, the coefficients ${\bar{\rho }}_{ij}$ can be calculated from the prescribed values of $\rho_{ij}$ [90,91].

### 5.2. Importance Measures of Parameters

The FORM formulation has an interesting feature because it provides the so called importance and sensitivity measures which basically tell us how important are the individual random variables compared to the other random variables. To have a broader context of how these importance measures result from the FORM method, a brief derivation (after Hohenbichler and Rackwitz [87], Bjerager and Krenk [88], Haukaas and Der Kiureghian [89], Der Kiureghian [90]) is presented below.

Assuming that $g_{u1}\left(u\right)=∥\nabla g_{u}∥\left(\beta -\alpha u\right)$ is the linearised limit-state function, noticing that the mean value of u equals zero, and its covariance is a unit matrix, the mean and variance of $g_{u1}\left(u\right)$ can be determined from

(14) $$\mu_{g_{u1}}=∥\nabla g_{u}∥\beta$$

(15) $$\sigma_{g_{u1}}^{2}={∥\nabla g_{u}∥}^{2}\left(\alpha_{1}^{2}+\alpha_{1}^{2}+\ldots +\alpha_{n}^{2}\right)={∥\nabla g_{u}∥}^{2}$$

where $\alpha =\nabla g_{u}/∥\nabla g_{u}∥$ is a unit vector, and $\beta =\mu_{g_{u1}}/\sigma_{g_{u1}}=\alpha u^{*}$ is the reliability index of the linearised problem. Moreover, Equation (15) shows that the components $\alpha_{i}^{2}$ are a function of the contribution of random variable $u_{i}$ to the total variance of the linearised limit-state function. It should be noted that the more significant the contribution, the more important is the influence of the random variable $u_{i}$. In this way, the elements of α give relative measures of importance for a standard normal variable $u_{i}$. In addition, analysing the expanded form of $g_{u1}\left(u\right)=∥\nabla g_{u}∥\left(\beta -\alpha_{1}u_{1}-\ldots -\alpha_{n}u_{n}\right)$, it should be noted that the positive value indicates that the random variable has a loading nature while the negative value points to a nature of resistance.

If the main random variables in the reliability problem are statistically independent, there exist reciprocal correspondence between the initial random variables $x_{i}$ and standard normal random variables $u_{i}$. Thus, the importance order and nature of random variables (loading or resistance) are similar to corresponding $u_{i}$ and can be obtained with respect to the vector α. If, however, the random variables x are statistically dependent, then there is no reciprocal correspondence. In such a situation, the vector α does not give information on the relative importance of the initial random variables. Therefore, to determine the measures of importance of the random variables, a linearised transformation is defined at the design point $u^{*}$

(16) $$u=u^{*}+J_{u,x}\left(\hat{x}-x^{*}\right)$$

where $\hat{x}$ can be regarded as a counterpart of x at the design point and differs inconsiderably from the vector x while the covariance matrix of u can be written as $\hat{\Sigma }=J_{u,x}^{-1}{\left(J_{u,x}^{-1}\right)}^{T}$. Using Equation (16) in the formula for $g_{u1}\left(u\right)$ and taking into account that $\beta =\alpha u^{*}$ leads to $g_{u1}\left(u\right)=-∥\nabla g_{u}∥\alpha J_{u,x}\left(\hat{x}-x^{*}\right)$. Hence, the variance of $g_{u1}\left(u\right)$ can be expressed as

(17) $$\sigma_{g_{u1}}^{2}={∥\nabla g_{u}∥}^{2}\left(\alpha J_{u,x}\hat{\Sigma }J_{u,x}^{T}\alpha^{T}\right)={∥\nabla g_{u}∥}^{2}\left({∥\alpha J_{u,x}\hat{D}∥}^{2}\alpha J_{u,x}\left(\hat{\Sigma }-\hat{D}\hat{D}\right)J_{u,x}^{T}\alpha^{T}\right)$$

where $\hat{D}=\mathrm{diag}\left[\hat{\sigma_{i}}\right]$ is a diagonal matrix of standard deviations of the vector $\hat{x}$ (e.g., Der Kiureghian [90]). The first component in the above expression includes the contributions to the variance $g_{u1}\left(u\right)$ which result from the individual element variances $\hat{x}$, while the second component represents the contributions resulting from the covariances of random variable pairs. Finally, a vector representing the relative importance of the random variables x can be defined

(18) $$\gamma =\alpha J_{u,x}\hat{D}.$$

which is usually normalised to a unit vector.

It can be proved that when the random variables are statistically independent, vector γ takes the form of vector α. Again, the formulas and the algorithm described in this section have been implemented in the OpenSees software framework [89,99] which is used in the calculations of the importance measures for the parameters of the analysed two-span beam.

### 5.3. Results of the Sensitivity Analysis

This section presents the results of sensitivity of input parameters on the nonlinear static response when applying to the vertical pushover analysis. Selected material and geometric parameters are treated as random variables with specified probability distribution, mean value, standard deviation or coefficient of variation. These probability properties are established based on recommended values found in literature (e.g., [85,89,103]). However, it should be noted that the assumed probability properties can differ and be updated if more accurate information is available.

The main concrete parameters subjected to probabilistic analysis are: compressive strength $f_{c0}$, ultimate compressive strength $f_{cu}$ and their associated strains $\varepsilon_{c0}$ and $\varepsilon_{cu}$. In this example, it is assumed that the mean values of these quantities are their nominal values. Therefore, for the compressive strength $f_{c0}$, the coefficient of variation equals cov=0.1 which results in the standard deviation σ=1.06MPa. Similarly, it is assumed that for $f_{cu}$, $\varepsilon_{c0}$ and $\varepsilon_{cu}$, the coefficient of variation is equal to 0.1. The coefficients of variation of the reinforcement bars $f_{y}$ and elastic modulus $E_{s}$ for steel are equal to 0.05 and 0.085, respectively. The area of each steel rebar is characterised by the coefficient of variation 0.01. The second group of uncertainty is related to both cross-section geometry and nodal coordinates. Uncertainty related to the concrete cross-section width and depth (height) is assumed in the standard deviation equal to 0.01m which for the section width *b* and height *h* results in the coefficients of variation equal to 0.05 and 0.025, respectively. The standard deviation of the cover thickness is equal to 0.01m which results in a relatively large coefficient of variation of 0.25. It is also assumed that all nodes have standard deviations 0.01m from their nominal *x* and *y* coordinates. The summary of the uncertainty parameters and their probability distributions are given in Table 1. In this study, the overall number of material and geometric parameters mapped to random variables equals 28.

As described in Section 5.2, the first-order reliability method has a useful side-effect which allows us to investigate the importance of individual parameters in a particular loading scenario. In this Section, the first-order reliability method is applied to the vertical pushover analysis. The problem is formulated as follows: what is the probability to exceed a specified threshold value of vertical displacement at node B when several material and structural parameters are treated as random variables (see Table 1). The limit-state function is specified as: $g=0.072-U_{B}$. However, unlike the regular reliability analysis using FORM, in this study, the probability of failure is not as interesting as the importance measures.

First it is assumed that all random variables are uncorrelated and, after running the first-order reliability analysis, the following importance measures in the form of the components of the vector γ (see Equation (18)) are presented in the 4th column of Table 2.

If assuming that the random variables for the two bay elements are correlated with the correlation coefficients specified in Table 1, then the ranking of parameters can be found in the 5th column of Table 2. From this table, we can observe that the correlation does not change the order of the ranking and only slightly changes the values of γ and corresponding percentage contribution ($\gamma^{2}\times 100\%$). Therefore, looking at columns 4 and 5, we can find that the most sensitive parameters are the cover thickness in element 1 (1st position: 43–44%) and element 2 (3rd position: 15–16%). This is mainly due to a relatively large coefficient of variation. Moreover, very high in the ranking is the depth *h* of element 1 (2nd position: 18%) and element 2 (5th position: 6%) because this parameter is part of flexural rigidity ($I=bh^{3}/12$). Next in the ranking, we can find the steel yield stress $f_{y}$ for element 1 (4th position: 8%) and element 2 (8th position: 1%), followed by the modulus of elasticity $E_{s}$ of element 1 (6th position: 4%) and element 2 (7th position: 2%). It is interesting that the concrete compressive strength $f_{c}$ is at 9th and 10th positions (less than 1%) in the ranking. The positive values of γ mean that the parameter plays the role of resistance, while the negative, the role of loading.

On the other hand, we assume that only one random variable is assigned to both elements 1 and 2, and the obtained results are presented in Table 3. This table summarises the averaged importance of parameters regardless of their location in the structure. The most important parameter for the whole structure is the concrete cover thickness followed by the concrete section depth *h*, the steel yield stress $f_{y}$, the elastic modulus of steel $E_{s}$ and the compressive concrete strength $f_{c0}$ with relative contributions of 59%, 24.4%, 7.6%, 6.6% and 1.2%, respectively. While all other parameters (including the concrete strain $\varepsilon_{c0}$, the concrete section width *b*, the bottom and top reinforcement areas $A_{s}$, the *X* and *Y* coordinates and the concrete ultimate stress and strain) have a contribution of less than 2%.

It is interesting to note that similar investigations on the importance measures for steel and reinforced concrete structures have been published in Haukaas and Der Kiureghian [89] and Haukaas and Scott [85]; however, they deal with the classic horizontal pushover analysis as opposed to the vertical one performed in this study. For instance, in Haukaas and Der Kiureghian [89], the most important parameters were horizontal loads, compressive strength and imperfections in horizontal nodal coordinates, whereas in Haukaas and Scott [85], the highest importance measures are related to concrete cross-section heights, the yield stress of rebar steel and a cross-section concrete cover. Nevertheless, direct comparison between these studies cannot be made mainly due to different model assumptions and probability characteristics. The author’s research plans include a comparison of importance measures of a nonlinear inelastic frame model under horizontal (seismic) and vertical (progressive collapse) pushover analyses.

## 6. Conclusions

A parametric analysis of the effects of a sudden support removal of a two-span reinforced concrete beam has been presented. Linear and nonlinear dynamic responses are analysed in detail and supported by vertical pushover computations and reliability-based sensitivity studies.

In the linear dynamic analysis, from the study of the influence of support removal rate, it can be concluded that the time of support removal has an important role when it is up to 30% of the first natural period of the beam without the inner support. In this case, the support removal can be regarded as ”sudden”, and it still generates substantial dynamic effects (dynamic factor of about 1.8). Moreover, the plot of the maximum vertical displacement versus the time of the support removal (response spectrum) presents a characteristic pattern (Figure 6) where the value of maximum vertical displacement is equal to the static vertical displacement when the time of support removal is the multiple of the first natural period. The maximum dynamic factor for the shortest time of support removal reaches the value of 1.8 when (5%) damping is applied in dynamic calculations.

When the material nonlinearity is included in the analysis, the characteristic pattern in the linear response spectrum is less pronounced and can be explained by the following facts: (a) in the nonlinear analysis, the stiffness contributing to the mode shape is calculated according to the current tangent stiffness and thus is responsible for the constant change in natural frequencies, which in turn desynchronise the contribution of mode shapes to the total structural dynamic response; (b) in the nonlinear cyclic analysis, at each deformation and loading cycle, the energy is dissipated providing additional damping to the assumed viscous damping and further influences the current natural frequencies and mode shapes (see, e.g., Rodrigues et al. [104]).

The vertical pushover analysis has been adopted from the horizontal pushover analysis from earthquake engineering. Four cases (one, two, and four concentrated loads and uniformly distributed load) of reference vertical load schemes have been considered to obtain the capacity curves of the vertical loading versus the vertical displacement at the point of the removed support. Moreover, the bending moments have been monitored at three critical nodes (the left fixed support, the midspan of the resulting one span beam, and the node of the removed support). It has been observed that the largest vertical capacity is obtained for the uniformly distributed load (100%), while for “4P”, “2P” and “1P”, the capacity is reduced to 80%, 60% and 51%, respectively. It is also noted that the failure mechanism depends on the vertical load distribution, and for the case “1P”, the first plastic hinge occurs at the point of removed support, while for “2P”, “4P” and “q” cases it occurs at the left fixed support which is related to the bending moment distributions.

It is also noted that comparing the current study to the studies of widespread subassemblies, ( e.g., Bao et al. [51], Alashker et al. [52], Lew et al. [78,79], Bao et al. [80]) representing an extracted fragment larger frames; in the case of two-span beam with one the roller support, (1) no arch effect is observed, (2) plastic hinges develop in discrete points equal to the number of the degree static indeterminacy and (3) the catenary action is only reflected by large vertical deformation and horizontal movement of the roller support.

A sensitivity analysis based on the importance measures of the first-order reliability method has been performed, and the result of all material and geometric parameters of the model has been presented using specified probability properties (probability distribution, mean and standard deviation). First, the sensitivity analysis results of the correlated and uncorrelated random variables are similar. Second, the first span parameters have higher sensitivity than the parameters of the second span; when only one parameter is specified for both spans, the most sensitive parameters when modelling and analysing the two-span beam of this paper are the geometrical dimensions of the cross-section, followed by the steel mechanical properties, while other parameters play a minor role in the sensitivity analysis. Note also that these first four group of parameters represent more than 97% of the total contribution of all parameters.

The assumption of the boundary conditions can be further investigated by changing the clamped support to a pinned one and adding rotational spring with its rotational stiffness factor $k_{\varphi }$. This additional parameter can be treated as random and can be incorporated in the sensitivity analysis which can be a subject of further investigations.

The results of this paper can help in planning experimental and numerical analyses of more complicated systems susceptible to progressive collapse.

It should also be emphasised that the results presented in this study, containing hundreds of finite element simulations, can be compared to the analyses using more sophisticated continuum-type finite elements (micro-modelling), which, however, will pose other modelling challenges such as computer memory and analysis time consumption. Moreover, a validation experimental test would be of interest; however, depending on the number of analysed support/column removal durations, it can face challenging, prohibitive costs or alternatively only selected representative cases could be validated.

## Figures and Tables

**Table 1:** Quantities mapped to random variables and their probabilistic properties (legend: PDF—probability density function: N—normal, LN—lognormal).

Quantity	PDF	Mean μ	Unit	Std. Dev. σ	cov	corr. ρ
*b*	N	2.0 × 10^−1^	m	1.0 × 10^−2^	0.050	0.6
*h*	N	4.0 × 10^−1^	m	1.0 × 10^−2^	0.025	0.6
$A_{s,top}$	N	2.011 × 10^−4^	m^2^	2.011 × 10^−6^	0.010	0.6
$A_{s,bottom}$	N	2.011 × 10^−4^	m^2^	2.011 × 10^−6^	0.010	0.6
cover	N	4.0 × 10^−2^	m	1.0 × 10^−2^	0.250	0.6
$E_{s}$	LN	2.0 × 10^8^	kPa	1.0 × 10^7^	0.050	0.6
$f_{y}$	LN	5.0 × 10^5^	kPa	4.25 × 10^4^	0.085	0.6
$f_{c0}$	LN	−1.06 × 10^4^	kPa	1.06 × 10^3^	0.100	0.6
$f_{cu}$	LN	−2.12 × 10^3^	kPa	2.12 × 10^2^	0.100	0.6
$\varepsilon_{c0}$	LN	−2.0 × 10^−3^	–	2.0 × 10^−4^	0.100	0.6
$\varepsilon_{cu}$	LN	−3.5 × 10^−3^	–	3.5 × 10^−4^	0.100	0.6
coord *Y*	N	0.0	m	1.0 × 10^−2^	–	0.0
coord *X*	N	0.0	m	1.0 × 10^−2^	–	0.0

**Table 2:** Ranking of parameters (uncorrelated and correlated random variables) according to the FORM importance measures and respective percentage contribution.

Order	Object	Parameter	$\gamma_{\mathrm{uncorrelated}}$ (in %)	$\gamma_{\mathrm{correlated}}$ (in %)	Nature
1	element 1	cover	0.6602660 (43.6)	0.6542139 (42.8)	loading
2	element 1	*h*	−0.4220153 (17.8)	−0.4189089 (17.5)	resistance
3	element 2	cover	0.3856375 (14.9)	0.3949241 (15.6)	loading
4	element 1	$f_{y}$	−0.2793387 (7.8)	−0.2785699 (7.8)	resistance
5	element 2	*h*	−0.2490824 (6.2)	−0.2551549 (6.5)	resistance
6	element 1	$E_{s}$	−0.2078843 (4.3)	−0.2066293 (4.3)	resistance
7	element 2	$E_{s}$	−0.1407877 (2.0)	−0.1435248 (2.1)	resistance
8	element 2	$f_{y}$	−0.0967715 (0.9)	−0.0979030 (1.0)	resistance
9	element 1	$f_{c0}$	−0.0899555 (0.8)	−0.0888733 (0.8)	resistance
10	element 2	$f_{c0}$	−0.0584756 (0.3)	−0.0601781 (0.4)	resistance
11	element 1	*b*	−0.0536994 (0.3)	−0.0521737 (0.3)	resistance
12	element 1	$\varepsilon_{c0}$	−0.0493609 (0.2)	−0.0512620 (0.3)	resistance
13	element 1	$A_{s,bottom}$	−0.0396915 (0.2)	−0.0392707 (0.2)	resistance
14	element 2	$\varepsilon_{c0}$	−0.0359152 (0.1)	−0.0365294 (0.1)	resistance
15	element 1	$A_{s,top}$	−0.0348488 (0.1)	−0.0349260 (0.1)	resistance
16	element 2	$A_{s,bottom}$	−0.0331608 (0.1)	−0.0337414 (0.1)	resistance
17	element 2	*b*	−0.0319813 (0.1)	−0.0333882 (0.1)	resistance
18	node 3	coord *X*	0.0305190 (0.1)	0.0307545 (0.1)	loading
19	node 1	coord *X*	−0.0272587 (0.1)	−0.0273524 (0.1)	resistance
20	element 2	$A_{s,top}$	−0.0064258 (0.0)	−0.0065277 (0.0)	resistance
21	node 2	coord *X*	−0.0032602 (0.0)	−0.0034021 (0.0)	resistance
22	node 2	coord *Y*	−0.0010419 (0.0)	−0.0010459 (0.0)	resistance
23	node 1	coord *Y*	0.0006312 (0.0)	0.0006296 (0.0)	loading
24	node 3	coord *Y*	0.0004107 (0.0)	0.0004163 (0.0)	loading
25	element 1	$\varepsilon_{cu}$	0.0000120 (0.0)	0.0000000 (0.0)	loading
26	element 1	$f_{cu}$	0.0000013 (0.0)	0.0000000 (0.0)	loading
27	element 2	$f_{cu}$	0.0000000 (0.0)	0.0000000 (0.0)	–
28	element 2	$\varepsilon_{cu}$	0.0000000 (0.0)	0.0000000 (0.0)	–

**Table 3:** Ranking of parameters according to the FORM importance measures and respective percentage contribution (case 3).

Order	Object	Parameter	γ (in %)	Nature
1	element 1 and 2	cover	0.7682050 (59.0)	loading
2	element 1 and 2	*h*	−0.4935972 (24.4)	resistance
3	element 1 and 2	$f_{y}$	−0.2752732 (7.6)	resistance
4	element 1 and 2	$E_{s}$	−0.2564705 (6.6)	resistance
5	element 1 and 2	$f_{c0}$	−0.1091943 (1.2)	resistance
6	element 1 and 2	$\varepsilon_{c0}$	−0.0643339 (0.4)	resistance
7	element 1 and 2	*b*	−0.0625824 (0.4)	resistance
8	element 1 and 2	$A_{s,bottom}$	−0.0534196 (0.3)	resistance
9	element 1 and 2	$A_{s,top}$	−0.0303648 (0.1)	resistance
10	node 3	coord *X*	0.0225389 (0.1)	loading
11	node 1	coord *X*	−0.0200180 (0.0)	resistance
12	node 2	coord *X*	−0.0025209 (0.0)	resistance
13	node 2	coord *Y*	−0.0007656 (0.0)	resistance
14	node 1	coord *Y*	0.0004599 (0.0)	loading
15	node 3	coord *Y*	0.0003057 (0.0)	loading
16	element 1 and 2	$f_{cu}$	0.0000000 (0.0)	resistance
17	element 1 and 2	$\varepsilon_{cu}$	0.0000000 (0.0)	resistance

## Data Availability

The data presented in this study are available on request.

## References

[1] Pearson C., Delatte N. Lessons from the Progressive Collapse of the Ronan Point Apartment Tower. Proceedings of the Third Forensic Engineering Congress.

[2] Osteraas J.D. (2006). Murrah Building Bombing Revisited: A Qualitative Assessment of Blast Damage and Collapse Patterns. J. Perform. Constr. Facil..

[3] Kazemi-Moghaddam A., Sasani M. (2015). Progressive collapse evaluation of Murrah Federal Building following sudden loss of column G20. Eng. Struct..

[4] Bazant Z.P., Verdure M. (2007). Mechanics of Progressive Collapse: Learning from World Trade Center and Building Demolitions. J. Eng. Mech..

[5] Mohamed O.A. (2006). Progressive Collapse of Structures: Annotated Bibliography and Comparison of Codes and Standards. J. Perform. Constr. Facil..

[6] Nair R.S. (2006). Preventing Disproportionate Collapse. J. Perform. Constr. Facil..

[7] Kokot S. (2009). Literature Survey on Current Methodologies of Assessment of Building Robustness and Avoidance of Progressive Collapse.

[8] Kokot S., Solomos G. (2012). Progressive Collapse Risk Analysis: Literature Survey, Relevant Construction Standards and Guidelines.

[9] Adam J.M., Parisi F., Sagaseta J., Lu X. (2018). Research and practice on progressive collapse and robustness of building structures in the 21st century. Eng. Struct..

[10] Kokot S., Anthoine A., Negro P., Solomos G. (2010). Static and Dynamic Analysis of a Reinforced Concrete Flat Slab Frame Building for Progressive Collapse.

[11] Kokot S., Anthoine A., Negro P., Solomos G. (2012). Static and dynamic analysis of a reinforced concrete flat slab frame building for progressive collapse. Eng. Struct..

[12] Dudziak S. (2021). Numerically Efficient Three-Dimensional Model for Non-Linear Finite Element Analysis of Reinforced Concrete Structures. Materials.

[13] Paulay T., Priestley M.J.N. (1992). Seismic Design of Reinforced Concrete and Masonry Buildings.

[14] Ellingwood B.R. (2006). Mitigating Risk from Abnormal Loads and Progressive Collapse. J. Perform. Constr. Facil..

[15] Grierson D.E., Xu L., Liu Y. (2005). Progressive-Failure Analysis of Buildings Subjected to Abnormal Loading. Comput.-Aided Civ. Infrastruct. Eng..

[16] Zhang L., Wei T., Li H., Zeng J., Deng X. (2021). Effects of Corrosion on Compressive Arch Action and Catenary Action of RC Frames to Resist Progressive Collapse Based on Numerical Analysis. Materials.

[17] Zhou H., Zhang Y., Fu F., Wu J. (2020). Collapse Mechanism of Single-Layer Cylindrical Latticed Shell under Severe Earthquake. Materials.

[18] Montuori R., Muscati R. (2017). Smart and simple design of seismic resistant reinforced concrete frame. Compos. Part B.

[19] Abarkane C., Rescalvo F.J., Donaire-Ávila J., Galé-Lamuela D., Benavent-Climent A., Molina A.G. (2018). Temporal Acoustic Emission Index for Damage Monitoring of RC Structures Subjected to Bidirectional Seismic Loadings. Materials.

[20] Neville A.M. (2011). Properties of Concrete.

[21] Kent D.C., Park R. (1971). Flexural members with confined concrete. J. Struct. Div..

[22] Scott B.D., Park R., Priestley M.J.N. (1982). Stress-strain behavior of concrete confined by overlapping hoops at low and high strain rates. ACI J..

[23] Mander J.B., Priestley M.J.N., Park R. (1988). Observed stress-strain model of confined concrete. J. Struct. Eng..

[24] Galeota D., Giammatteo M.M., Marino R. Strength and ductility of confined high strength concrete. Proceedings of the Earthquake Engineering, Tenth World Conference.

[25] Mainstone R. (1975). Properties of materials at high rates of straining or loading. Mater. Struct..

[26] Asprone D., Frascadore R., Ludovico M.D., Prota A., Manfredi G. (2012). Influence of strain rate on the seismic response of RC structures. Eng. Struct..

[27] Filiatrault A., Holleran M. (2001). Stress-strain behavior of reinforcing steel and concrete under seismic strain rates and low temperatures. Mater. Struct..

[28] Comite Euro-International du Beton (1988). Concrete structures under impact and impulsive loading. CEB Bulletin.

[29] Comite Euro-International du Beton (1990). Model Code. CEB Bulletin.

[30] Malvar L.J., Crawford J.E. Dynamic increase factors for concrete. Proceedings of the Twenty-Eighth DDESB Seminar.

[31] Fu H.C., Erki M.A., Seckin M. (1991). Review of effects of loading rate on concrete in compression. J. Struct. Eng..

[32] Bischoff P.H., Perry S.H. (1991). Compressive behaviour of concrete at high strain rates. Mater. Struct..

[33] Malvar L.J. (1998). Review of static and dynamic properties of steel reinforcing bars. ACI Mater. J..

[34] Mander J.B., Priestley M.J.N., Park R. (1988). Theoretical stress-strain model for confined concrete. J. Struct. Eng..

[35] Guedes J., Pegon P., Pinto A.V. (1994). A Fibre Timoshenko Beam Element in Castem 2000.

[36] Braga F., Gigliotti R., Laterza M. (2006). Analytical Stress–Strain Relationship for Concrete Confined by Steel Stirrups and/or FRP Jackets. J. Struct. Eng..

[37] D’Amato M., Braga F., Gigliotti R., Kunnath S., Laterza M. (2012). A numerical general-purpose confinement model for non-linear analysis of R/C members. Comput. Struct..

[38] Hu D. (2012). Efficient Finite Element Modeling of Reinforced Concrete Columns Confined with Fiber Reinforced Polymers. Ph.D. Thesis.

[39] Comite Euro-International du Beton (2010). Model Code for Concrete Structures.

[40] Martinez-Rueda J.E., Elnashai A.S. (1997). Confined concrete model under cyclic load. Mater. Struct..

[41] Derkowski W., Walczak R. (2021). Possibilities of Increasing Effectiveness of RC Structure Strengthening with FRP Materials. Materials.

[42] Kozielova M., Marcalikova Z., Mateckova P., Sucharda O. (2020). Numerical Analysis of Reinforced Concrete Slab with Subsoil. Civ. Environ. Eng..

[43] Limkatanyu S., Spacone E. Nonlinear analysis of reinforced concrete frames including bond-slip effects. Proceedings of the 14th World Conference on Earthquake Engineering.

[44] Kenyon J.M., Warner R.F. (1992). Refined Analysis of Nonlinear Behaviour of Concrete Structures.

[45] Filippou F.C., Issa A. (1988). Nonlinear Analysis of Reinforced Concrete Frames under Cyclic Load Reversals.

[46] Oller S., Oñate E., Oliver J., Lubliner J. (1990). Finite element nonlinear analysis of concrete structures using a plastic-damage model. Eng. Fract. Mech..

[47] Praxedes C., Yuan X. (2021). Robustness-oriented optimal design for reinforced concrete frames considering the large uncertainty of progressive collapse threats. Struct. Saf..

[48] Bao Y., Main J., Lew H., Sadek F. Robustness Assessment of RC Frame Buildings under Column Loss Scenarios. Proceedings of the Structures Congress.

[49] Mucedero G., Brunesi E., Parisi F. (2021). Progressive collapse resistance of framed buildings with partially encased composite beams. J. Build. Eng..

[50] Main J., Bao Y., Lew H., Sadek F. Robustness of Precast Concrete Frames: Experimental and Computational Studies. Proceedings of the Structures Congress.

[51] Bao Y., Kunnath S.K., El-Tawil S., Lew H.S. (2008). Macromodel-Based Simulation of Progressive Collapse: RC Frame Structures. J. Struct. Eng..

[52] Alashker Y., Li H., El-Tawil S. (2011). Approximations in Progressive Collapse Modeling. J. Struct. Eng..

[53] Neuenhofer A., Filippou F.C. (1997). Evaluation of nonlinear frame finite element models. J. Struct. Eng..

[54] Calabrese A. (2008). Numerical Issues in Distributed Inelasticity Modelling of RC Frame Elements for Seismic Analysis. Ph.D. Thesis.

[55] McKenna F. (1999). Object-Oriented Finite Element Programming: Frameworks for Analysis, Algorithms and Parallel Computing. Ph.D. Thesis.

[56] McKenna F., Fenves G.L., Scott M.H. (2003). Opensees—Open System for Earthquake Engineering Simulation.

[57] Scott M.H., Fenves G.L., McKenna F., Filippou F.C. (2008). Software Patterns for Nonlinear Beam-Column Models. J. Struct. Eng..

[58] Welch B.B., Jones K., Hobbs J. (2003). Practical Programming in Tcl and Tk.

[59] Ousterhout J.K., Jones K. (2010). Tcl and the Tk Toolkit.

[60] Zhu M., McKenna F., H.Scott M. (2018). OpenSeesPy: Python library for the OpenSees finite element framework. Softw. X.

[61] Neuenhofer A., Filippou F.C. (1998). Geometrically nonlinear flexibility-based frame finite element. J. Struct. Eng..

[62] Menegotto M., Pinto P.E. (1973). Method of Analysis for Cyclically Loaded Reinforced Concrete Plane Frames Including Changes in Geometry and Non-Elastic Behavior of Elements under Combined Normal Force and Bending. IABSE Symposium on Resistance and Ultimate Deformability of Structures Acted on by Well Defined Repeated Loads.

[63] Filippou F.C., Popov E.P., Bertero V.V. (1983). Effects of Bond Deterioration on Hysteretic Behavior of Reinforced Concrete Joints.

[64] Bosco M., Ferrara E., Ghersi A., Marino E.M., Rossi P.P. Improvement of the model proposed by Menegotto and Pinto for steel. Proceedings of the Second European Conference on Earthquake Engineering and Seismology.

[65] Bosco M., Ferrara E., Ghersi A., Marino E.M., Rossi P.P. (2016). Improvement of the model proposed by Menegotto and Pinto for steel. Eng. Struct..

[66] Hognestad E. (1951). A Study of Combined Bending and Axial Load in Reinforced Concrete Members.

[67] Karsan I.D., Jirsa J.O. (1969). Behavior of concrete under compressive loading. J. Struct. Div..

[68] De Souza R.M. (2000). Force-Based Finite Element for Large Displacement Inelastic Analysis of Frames. Ph.D. Thesis.

[69] Crisfield M. (1991). Nonlinear Finite Element Analysis of Solids and Structures.

[70] Park R., Paulay T. (1975). Reinforced Concrete Structures.

[71] Espion B., Halleux P. (1988). Moment curvature relationship of reinforced concrete sectionsunder combined bending and normal force. Mater. Struct..

[72] Srikanth M., Kumar G.R., Giri S. (2007). Moment curvature of reinforced concrete beams using various confinement models and experimental validation. Asian J. Civ. Eng..

[73] Newmark N. (1959). A method of computation for structural dynamics. J. Eng. Mech. Div..

[74] Hilber H., Hughes T., Taylor R. (1977). Improved numerical dissipation for time integration algorithms in structural dynamics. Earthq. Eng. Struct. Dyn..

[75] Russell J.M., Owen J., Hajirasouliha I. (2015). Experimental investigation on the dynamic response RC flat slabs after a sudden column loss. Eng. Struct..

[76] Russell J.M., Owen J., Hajirasouliha I. (2019). Dynamic column loss analysis of reinforced concrete flat slabs. Eng. Struct..

[77] Adam J.M., Buitrago M., Sagaseta J., Moragues J.J. (2020). Dynamic performance of a real-scale reinforced concrete building test under a corner-column failure scenario. Eng. Struct..

[78] Lew H., Bao Y., Sadek F., Main J.A., Pujol S., Sozen M.A. (2011). An Experimental and Computational Study of Reinforced Concrete Assemblies under a Column Removal Scenario.

[79] Lew H.S., Bao Y., Pujol S., Sozen M.A. (2014). Experimental study of rc assemblies under a column removal scenario. ACI Struct. J..

[80] Bao Y., Lew H.S., Kunnath S.K. (2012). Modeling of Reinforced Concrete Assemblies under Column-Removal Scenario. J. Struct. Eng..

[81] Gu X., Zhanga B., Wang Y., Wang X. (2021). Experimental investigation and numerical simulation on progressive collapse resistance of RC frame structures considering beam flange effects. J. Build. Eng..

[82] Kleiber M., Antunez H., Hien T.D., Kowalczyk P. (1997). Parameter Sensitivity in Nonlinear Mechanics.

[83] Scott M.H. (2004). Software Frameworks for the Computational Simulation of Structural Systems. Ph.D. Thesis.

[84] Scott M.H., Franchin P., Fenves G.L., Filippou F.C. (2004). Response Sensitivity for Nonlinear Beam-Column Elements. J. Struct. Eng..

[85] Haukaas T., Scott M.H. (2006). Shape sensitivities in the reliability analysis of nonlinear frame structures. Comput. Struct..

[86] Scott M.H. (2012). Evaluation of Force-Based Frame Element Response Sensitivity Formulations. J. Struct. Eng..

[87] Hohenbichler M., Rackwitz R. (1986). Sensitivity and importance measures in structural reliability. Civ. Eng. Syst..

[88] Bjerager P., Krenk S. (1989). Parameter sensitivity in first order reliability theory. J. Eng. Mech..

[89] Haukaas T., Der Kiureghian A. (2005). Parameter sensitivity and importance measures in nonlinear finite element reliability analysis. J. Eng. Mech..

[90] Der Kiureghian A. (2004). First- and Second-Order Reliability Methods. Engineering Design Reliability Handbook.

[91] Liu P.L., Der Kiureghian A. (1986). Multivariate distribution models with prescribed marginals and covariances. Probabilistic Eng. Mech..

[92] Liu P.L., Der Kiureghian A. (1991). Optimization algorithms for structural reliability. Struct. Saf..

[93] Ditlevsen O., Madsen H.O. (2007). Structural Reliability Methods.

[94] Der Kiureghian A., Haukaas T., Fujimura K. (2006). Structural reliability software at the University of California, Berkeley. Struct. Saf..

[95] Haukaas T., Der Kiureghian A. (2004). Finite Element Reliability and Sensitivity Methods for Performance-Based Earthquake Engineering.

[96] Gu Q. (2014). Performance and Risk Assessment of Soil-Structure Interaction Systems Based on Finite Element Reliability Methods. Math. Probl. Eng..

[97] Der Kiureghian A. (1996). Structural reliability methods for seismic safety assessment: A review. Eng. Struct..

[98] Chang K.H. (2013). Reliability analysis. Product Performance Evaluation Using CAD/CAE.

[99] Scott M.H., Haukaas T. (2008). Software Framework for Parameter Updating and Finite-Element Response Sensitivity Analysis. J. Comput. Civ. Eng..

[100] Benjamin J.R., Cornell C.A. (1970). Probability, Statistics, and Decision for Civil Engineers.

[101] Nataf A. (1962). Determination des distribution don’t les marges sont donnees. Comptes Rendus L’Academic Sci..

[102] Rosenblatt M. (1952). Remarks on a multivariate transformation. Ann. Math. Stat..

[103] Chmielewski T., Konopka E. (1999). Statistical evaluations of field concrete strength. Mag. Concr. Res..

[104] Rodrigues H., Arede A., Varum H., Costa A.G. Energy dissipation and equivalent damping of RC columns subjected to biaxial bending: An investigation based in experimental results. Proceedings of the fifthteenth World Conference on Earthquake Engineering.

